# A novel non-sense variant in *GSDME* causing exon skipping associated with DFNA5 in a large Chinese family

**DOI:** 10.3389/fneur.2026.1752843

**Published:** 2026-02-06

**Authors:** Bingqian Yang, Mingwan Zhu, Xicui Long, Siwei Wan, Yu Lu, Huijun Yuan, Qingquan Hua

**Affiliations:** 1 Department of Otolaryngology-Head and Neck Surgery, Renmin Hospital of Wuhan University, Wuhan, Hubei, China; 2 Institute of Rare Diseases, West China Hospital of Sichuan University, Chengdu, Sichuan, China

**Keywords:** DFNA5, GSDME, hereditary hearing loss, phenotype variability, splicing analysis

## Abstract

**Background:**

Hereditary hearing loss demonstrates significant genetic heterogeneity, involving diverse genes and variation types. Autosomal dominant forms present particular challenges in variant interpretation due to variable expressivity.

**Objectives:**

This study aimed to clinically and molecularly characterize a multi-generational family with autosomal dominant hereditary hearing loss, and to functionally validate the pathogenicity of an identified novel variant.

**Methods:**

Comprehensive clinical evaluations included audiometric testing and medical history review. Genetic analysis employed whole-exome sequencing followed by Sanger validation. Functional characterization involved minigene splicing assays and transcript analysis to assess the impact on splicing mechanisms.

**Results:**

Affected individuals exhibited post-lingual, bilateral, symmetric, progressive sensorineural hearing loss, initially affecting high frequencies. We identified a novel *GSDME* mutation (NM_001127453.2:c.1123G>T; p.Glu375Ter) that disrupts an exon splicing enhancer, causing exon 8 skipping and frameshift alterations. Functional assays confirmed reduced enhancer activity and aberrant splicing. Literature review of 20 reported mutations revealed substantial phenotypic variability and highlighted limitations of splice prediction algorithms.

**Conclusions:**

Our findings expand the *GSDME* mutation spectrum and provide functional evidence supporting a pathogenic role for non-sense variants through splicing disruption mechanisms. This study reinforces the potential gain-of-function hypothesis for *GSDME*-associated hearing loss and emphasizes the necessity of functional validation for accurate variant interpretation.

## 1 Introduction

Hearing loss represents a major global health burden with a strong genetic basis, where inherited factors contribute to 40–60% of cases across a highly heterogeneous landscape of over 150 implicated genes (1). This heterogeneity poses a significant challenge in autosomal dominant non-syndromic hearing loss (ADNSHL), where variable expressivity and incomplete penetrance often complicate the interpretation of genetic variants.

Deafness autosomal dominant 5 (DFNA5), caused by mutations in the *GSDME* gene, stands out for its exceptionally same pathogenic mechanism. Since its initial linkage to chromosome 7p15 (2), research has revealed that virtually all pathogenic *GSDME* variants converge on a single molecular outcome: the complete skipping of exon 8 in the mRNA (3, 4). This results in a consistent, truncated protein product. The invariance of this splicing defect across diverse mutations, coupled with normal hearing in *GSDME*-knockout mice, provides compelling evidence for a gain-of-function (GOF) pathogenesis (5). Historically, haploinsufficiency mechanisms-related non-sense or frameshift variants of *GSDME* were thought to be benign (6).

This study describes a large Chinese ADNSHL pedigree and identified a novel, segregating non-sense variant in *GSDME* (c.1123G>T; p.Glu375Ter). Minigene assays confirmed this variant causes exon 8 skipping. Our finding challenges the traditional benign classification of *GSDME* non-sense variants, expands the mutational spectrum of DFNA5, and necessitates a revised mechanistic understanding of how PTCs can lead to GOF pathology.

## 2 Materials and methods

### 2.1 Subjects and clinical characterization

This study included a large Han Chinese pedigree with sensorineural hearing loss, consisting of 72 family members. Each member provided demographic information, age of onset, illness development, obstetric history, noise exposure, ototoxic medication use, head trauma, infectious infections, family history, and other pertinent clinical symptoms. Audiological assessments and otolaryngological examinations were conducted on probands, including pure-tone audiometry, impedance testing, distortion product otoacoustic emissions, auditory brainstem response, and temporal bone CT scans. Pure-tone audiometry was performed to assess the hearing of other family members using frequencies of 0.125 kHz, 0.25 kHz, 0.5 kHz, 1 kHz, 2 kHz, 4 kHz, and 8 kHz via air conduction bilaterally. The average values of the thresholds of air conduction were determined at 500–4,000 Hz to determine the degree of hearing loss in the family. The study protocol was approved by the Institutional Review Board of West China Hospital [2021 Audit (190)]. All family members provided written informed consent before participating in this study.

### 2.2 Whole-genome sequencing and bioinformatic analysis

Approximately 5 mL of peripheral blood was collected from the proband and family members. DNA was isolated using the AxyPrep-96 Blood Genomic DNA Kit (Axygen BioScience, Union City, CA, USA). Whole-genome sequencing (WGS) sequencing was performed on the proband (III-2) and another family member (VI-4) using DNBSEQ-T7 sequencing instruments (MGI, Shenzhen, China), with paired-end reads of 100 base pairs. The sequenced reads were aligned to the human reference genome (GRCh38/hg38) using the Burrows-Wheeler Aligner (BWA v0.7.10). Subsequent processing, including duplicate marking, local realignment, and base quality score recalibration, was performed using the Genome Analysis Toolkit (GATK v4.0). Variant calling was conducted with GATK HaplotypeCaller. The identified variants were filtered using the following criteria: (1) population frequency < 1% in the gnomAD and an in-house Chinese database; (2) presence in a custom panel of 189 genes known to be associated with hearing loss (Supplementary material); and (3) compatible with an autosomal dominant inheritance model. The functional impact of prioritized variants was predicted using PolyPhen-2, MutationTaster, and SIFT.

### 2.3 Segregation analysis Sanger sequencing

Sanger sequencing was used for variants validation. Primer3 online (https://www.bioinformatics.nl/cgi-bin/primer3plus/primer3plus.cgi) was used to design primers for the *GSDME* gene variants. A pair of exon 8 primers (forward, 5′-CCGTCAGTGAAATGTAGCC-3′ and reverse, 5′-TTCCACAGTTACCACCTCTG-3′) across the intron-exon boundaries were used to span the sequences. One hundred ng Genomic DNA (gDNA) was used as a template in a total reaction volume of 15 μL (2 × PrimeSTAR Max Premix 7.5 μl, primer forward 10 μmol/L 0.25 μL, primer reverse 10 μmol/L 0.25 μL, and dH2O to 15 μL). PCR was performed with an initial denaturation at 98 °C for 1 min, followed by 35 cycles of 98 °C for 10 s, 60 °C for 5 s, and 72 °C for 20 s, and a final extension at 72 °C for 5 min. Direct sequencing of the DNA in both directions was performed by Sangon Biotech (Chengdu, China). The reference sequence of *GSDME* used was GenBank NM_001127453.2.

### 2.4 *In vitro* splicing analysis

The pSPL3 vector, pre-purchased by our research group, was utilized to construct plasmids for *in vitro* splicing validation. This vector has 6031 base pairs and comprises an SV40 promoter, as well as two exons, SD6 and SA2. Specific primers with XhoI and BamHI restriction enzyme sites (F5′-CCCTCGAGCCGTCAGTGAAATGTAGCC3′ and R5′-CGGGATCCTTCCACAGTTACCACCTCTG3′) were utilized to amplify a 471 bp fragment that encompasses exon 8 of *GSDME*. The PCR products and plasmid underwent digestion with XhoI (Takara, 1094S) and BamHI (Takara, 1010S) restriction enzymes, respectively. Subsequently, T4 ligase (Takara, 2011A) was used for ligation. The ligated products were then transformed into E.coli DH5α Competent Cells (Takara, 9057) for amplification. Finally, single colonies were selected for sequencing to confirm the accuracy of the mutation.

The mini-gene vector was transfected into COS-7 cells using Lipofectamine 3000 (Invitrogen, L3000015). After 48 h, cells were collected and RNA was extracted using the Trizol (Invitrogen, 15596018). The extracted RNA was reverse transcribed into cDNA with a reverse transcription kit (Takara, RR047A). Amplification was performed using exon-specific primers (SD6-F: TCTGAGTCACCTGGACAACC and SA2-R: ATCTCAGTGGTATTTGTGAGC) from pSPL3. PCR was performed with an initial denaturation at 95 °C for 3 min, followed by 35 cycles of 95 °C for 15 s, 60 °C for 15 s, and 72 °C for 15 s, and a final extension at 72 °C for 5 min. The PCR products were electrophoresed on a 2% agarose gel. Direct sequencing of the DNA in both directions was performed by Sangon Biotech (Chengdu, China). Three controls were included in each transfection experiment: (1) the wild-type (WT) construct (negative control for aberrant splicing); (2) a construct carrying a known pathogenic splice-site variant (c.991-21_991-19delTTCinsT) (positive control for exon 8 skipping); and (3) the empty pSPL3 vector (background control).

### 2.5 Literature review and splicing prediction

A thorough evaluation was undertaken on pathogenic variations of *GSDME* previously identified in persons. PubMed was used to conduct literature searches using the keywords (*GSDME* OR DFNA5) AND (hearing loss OR deafness). The most recent search occurred on March 22, 2024. Information was collected on the location of the variant, age of onset, audiometric manifestations, associated symptoms, and other systemic abnormalities documented in the literature in affected individuals.

The MANE SELECT transcript was used to convert all filtered pathogenic variants. dbscSNV, MaxEntScan, and Splice AI were used to predict the effect of mutations on splicing. In addition, ESEfinder 3.0 (https://esefinder.ahc.umn.edu) was used for splicing prediction.

## 3 Results

### 3.1 Clinical funding

**Figure 1:**
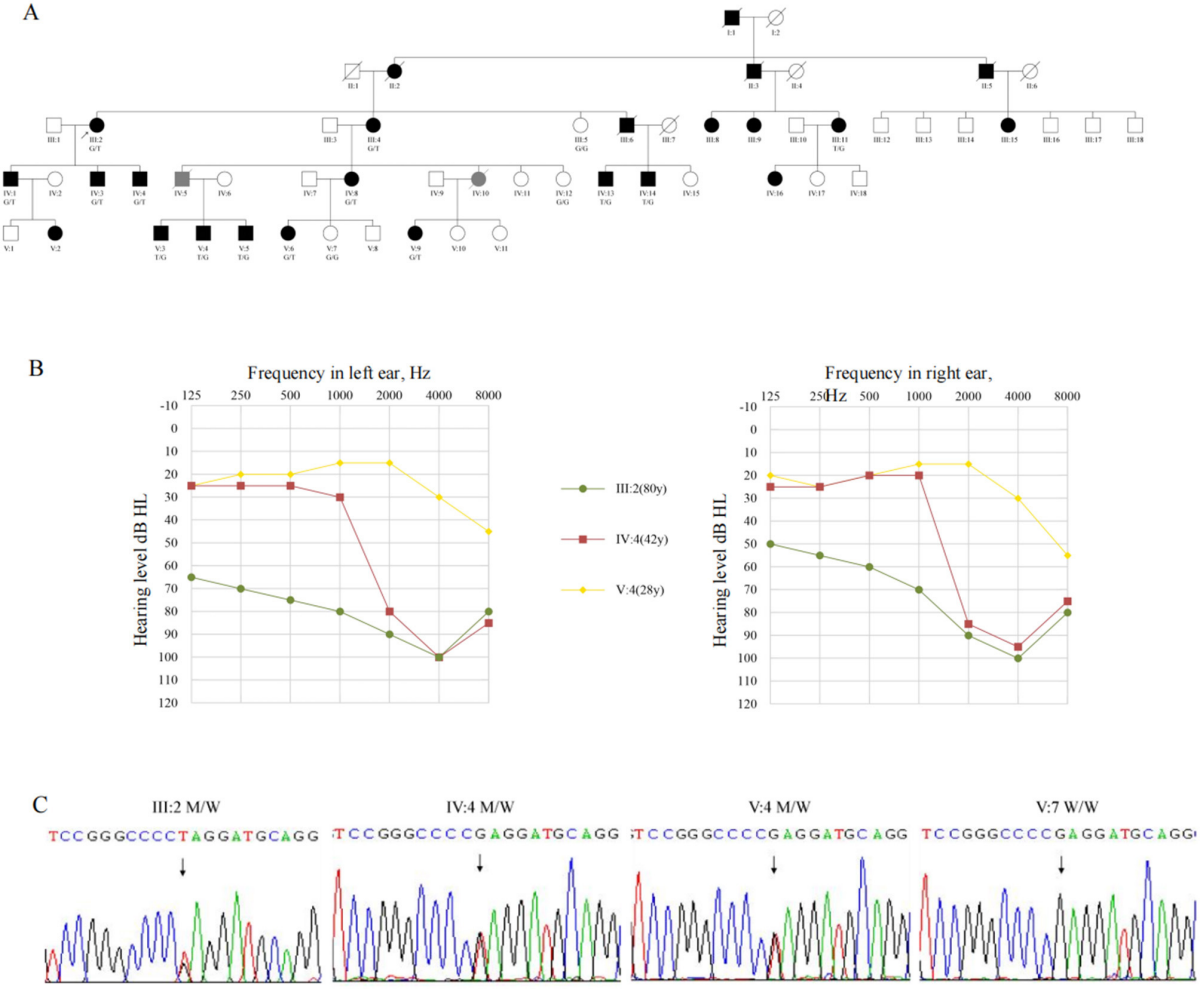
The pedigree presents autosomal dominant non-syndromic sensorineural deafness. **(A)** The pedigree of the family. 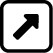, Proband; T, Mutation allele; G, Wild-type allele. **(B)** The pure tone audiometry of III:2, IV-4, V-4. **(C)** Sanger sequencing of III:2, IV-4, V-4, V-7. T, Mutation allele; G, Wild-type allele.

The pedigree(Figure 1A) shows a typical autosomal dominant inheritance pattern of hereditary hearing loss, covering five generations and including a total of 55 family members. There is no gender disparity among affected individuals in the pedigree. Hearing loss typically appears during the first or second decades of life. It is characterized by post-lingual, bilateral, symmetric, and progressive sensorineural deafness. Initially, it affects high frequencies and gradually extends to involve all frequencies (Figure 1B). Notably, individuals IV-1 and IV-3 present with asymmetric hearing loss due to otitis media. Most patients report intermittent tinnitus without symptoms of vertigo or balance disturbances and with normal intellectual and cognitive functions. None of the patients had a history of aminoglycoside exposure or noise exposure before presentation, and physical exams were normal. High-resolution CT scan of the temporal bone showed no abnormality. Thirteen patients showed moderate to severe bilateral sensorineural hearing loss during auditory testing. The audiograms showed that the high frequencies were affected at the beginning and that the middle and low frequencies were gradually affected with increasing age (Table 1). Although V-8 currently has average hearing thresholds within the normal range, the audiogram showed a significant decline in high-frequency hearing (Figure 1B).

**Table 1:** Summary of clinical data for family members.

**Subjects**	**Age of test (years)**	**Age of onset (years)**	**Nucleotide change**	**PTA-right (dB HL)**	**PTA-left (dB HL)**	**Tinnitus**	**Vertigo**
III:2	80	10+	c.1123G>T	80	86.25	Y	N
III:4	77	20+	c.1123G>T	87.5	88.75	Y	N
III:5	71	N	Wide type	32.5	43.75	N	N
III:11	70	20+	c.1123G>T	100	77.5	NA	NA
IV:1	57	20+	c.1123G>T	81.25	100	NA	NA
IV:3	49	19	c.1123G>T	68.75	100	Y	N
IV:4	42	19	c.1123G>T	55	58.75	Y	N
IV:8	55	15	c.1123G>T	87.5	92.5	Y	N
IV:12	44	N	Wide type	20	25	N	N
IV:13	43	18	c.1123G>T	80	77.5	Y	N
IV:14	40	10+	c.1123G>T	95	80	Y	N
V:3	30	10+	c.1123G>T	60	57.5	Y	N
V:4	28	20+	c.1123G>T	20	20	N	N
V:5	26	22	c.1123G>T	35	33.75	Y	N
V:6	29	23	c.1123G>T	63.75	69	Y	N
V:7	28	N	Wide type	15	12.5	N	N
V:9	24	18	c.1123G>T	55	58.75	Y	N

### 3.2 Identification of *GSDME* mutation

Whole-genome sequencing of the proband (III-2) and an unaffected relative (IV-4) initially yielded multiple variants in hearing loss-associated genes. After filtering against population databases and enforcing an autosomal dominant inheritance model, a single candidate variant emerged: a heterozygous c.1123G>T transition in exon 8 of *GSDME* (NM_001127453.2), predicted to introduce a premature termination codon (p.Glu375Ter). Sanger sequencing confirmed this variant co-segregated perfectly with the hearing loss phenotype in the family (Figure 1C) and was absent from gnomAD, 1,000 Genomes, and our in-house controls.”

“A second heterozygous variant in *MITF* (c.1156G>C, p.Val386Leu) was also identified in both sequenced individuals. However, it was excluded as causative due to phenotype mismatch (no features of Waardenburg syndrome) and lack of co-segregation within the pedigree.

### 3.3 Splicing analysis

**Figure 2:**
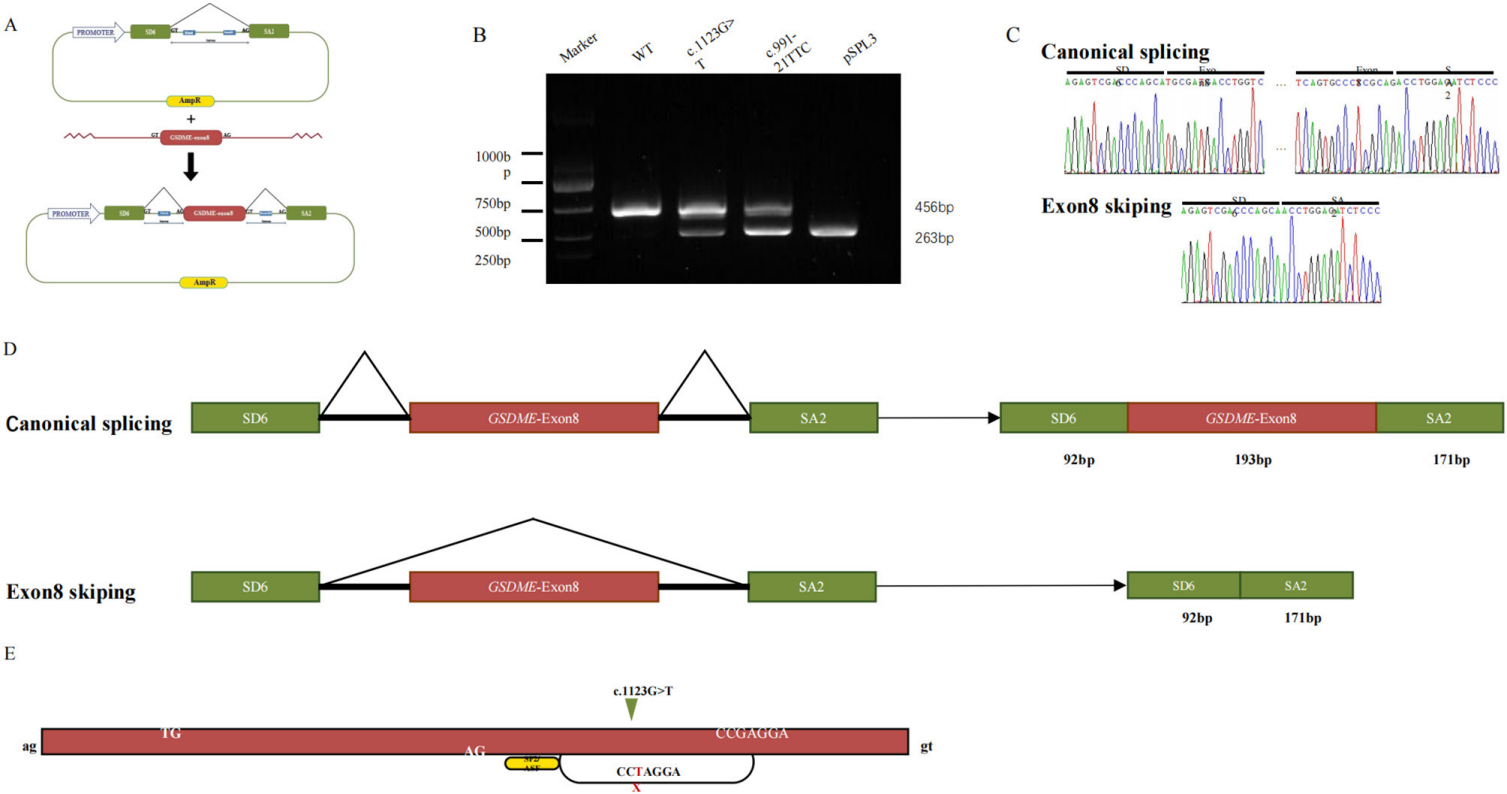
*In vitro* analysis of splice site variant. **(A)** Mini-gene splicing analysis and schematic representation of *GSDME* exon 8 and splicing factor binding. **(B)** Splicing-assay products were separated by agarose gel. **(C)** Sanger sequencing of the bands. **(D)** The schematic diagram of splicing patterns. **(E)** Predicted the ESE of the novel variant in this study, wild-type black and mutant red.

The *in vitro* minigene assay was employed to determine the effect of c.1123G>T on splicing. RT-PCR analysis of RNA from transfected COS-7 cells yielded a single 456-bp product for the wild-type construct, which sequencing confirmed contained the vector-derived SD6 exon, *GSDME* exon 8, and the SA2 exon (Figures 2B, C). In contrast, the mutant construct produced two products: the expected 456-bp wild-type band and a predominant aberrant band of 263 bp (Figure 2B). Sequencing of the smaller band confirmed the complete absence of *GSDME* exon 8, indicating direct splicing from SD6 to SA2 (Figures 2C, D). This exon skipping creates a frameshift, introducing a premature termination codon at position 372 and predicting a truncated protein lacking the C-terminal 126 amino acids.”

“Bioinformatic analysis using ESEfinder 3.0 suggested that the c.1123G>T substitution disrupts a binding motif for the splicing factor SF2/ASF, potentially reducing exonic splicing enhancer activity and contributing to exon 8 skipping (Figure 2E).

### 3.4 Phenotypic analysis and splice predictions of different variants

**Figure 3:**
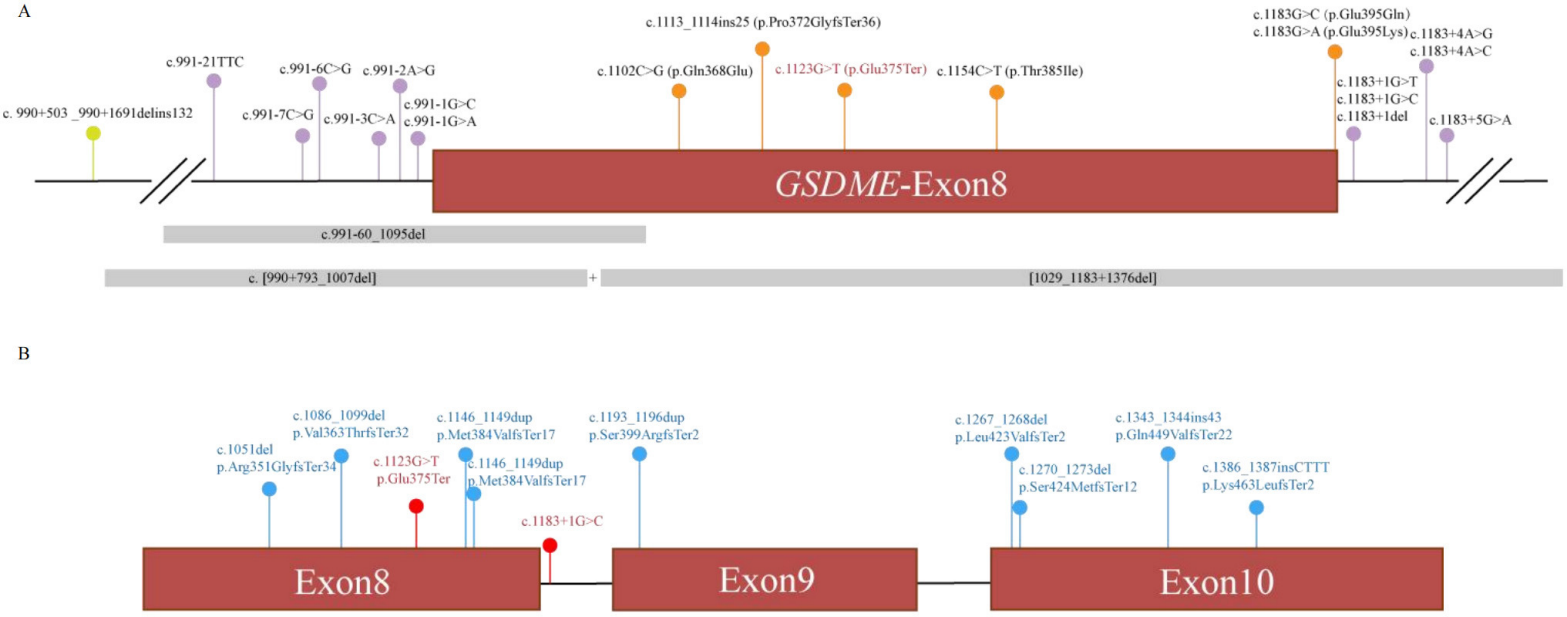
**(A)** Novel (red) and previously described (black) variants alignment to *GSDME* exon 8 and its flanking introns. Orange, variants in exon; purple, variants in splice region; green, variants in intron; gray, CNV; **(B)** LOF variants in GDC. Red, pathogenic variants; blue, begin variants.

A review of the literature identified 20 previously reported pathogenic GSDME variants, predominantly clustered around exon 8 (Figure 3A, Table 2). All experimentally tested variants have been shown to cause exon 8 skipping. The associated phenotypes consistently involve progressive, high-frequency sensorineural hearing loss with an onset ranging from 5 to 50 years, often accompanied by tinnitus. Considerable intra- and inter-familial phenotypic variability was noted, even for the same mutation.

**Table 2:** Summary of all reported *GSDME* variants leading to hearing loss.

**Mutation (NM_001127453.2)**	**Location**	**Effect**	**Age of onset (years)**	**PTA**	**Tinnitus**	**Vertigo**	**Country**	**dbscSNV**	**MaxEntScan**	**Splice AI**	**References**
c.991-60_1095del	Intron 7-Exon 8	Exon 8 skipping	6–20	Sloping	NA	NA	France	NA	NA	+	(17)
c.[990+793_1007del; 1029_1183+1376del]	Intron 7-Intron 8	Exon 8 skipping	6	Sloping to flat	NA	NA	France	NA	NA	NA	(17)
c.990+503_990+1691delins132	Intron 7	Exon 8 skipping	5–15	Sloping	NA	NA	Dutch	NA	NA	NA	(14)
c.991-15_991-13del	Intron 7	Exon 8 skipping	20–31	Sloping to flat	Y	–	China	NA	NA	–	(15)
			20	Sloping to flat	NA	–	Korea	NA	NA	–	(18)
			7–30	Sloping	NA	NA	China	NA	NA	–	(19)
			20–35	Sloping	NA	NA	China	NA	NA	–	(20)
			17–21	Cookie-bite	NA	NA	China	NA	NA	–	(20)
			17	Sloping	NA	NA	China	NA	NA	–	(20)
			25–35	Sloping to flat	Y	–	China	NA	NA	–	(21)
			10–20	Sloping	NA	NA	USA	NA	NA	–	(3)
			6–13	flat	NA	NA	USA	NA	NA	–	(15)
c.991-7C>G	Intron 7	Exon 8 skipping	10	Sloping	NA	NA	China	–	+	–	(20)
c.991-6C>G	Intron 7	Exon 8 skipping	0–40	Sloping	NA	NA	Dutch	–	+	–	(22)
c.991-3C>A	Intron 7	Exon 8 skipping	20–39	Sloping	Y	-	China	+	+	–	(15)
c.991-2A>G	Intron 7	Exon 8 skipping	13–61	Sloping to flat	NA	NA	China	+	+	+	(23)
			10–20	Sloping	NA	NA	USA	+	+	+	(3)
			8–18	Sloping	NA	NA	China	+	+	+	(24)
c.991-1G>C	Intron 7	Exon 8 skipping	20–40	Sloping to flat	NA	NA	China	+	+	+	(25)
			18–30	Cookie-bite	NA	NA	China	+	+	+	(20)
c.1102C>G	Exon 8	Exon 8 skipping	10–20	Sloping to flat	NA	NA	USA	NA	NA	–	(3)
c.1113_1114ins25	Exon 8	Frameshift variant	7–30	Sloping to flat	NA	–	China	NA	NA	–	(26)
c.1123G>T	Exon 8	Exon 8 skipping	10–30	Sloping	Y	N	China	NA	NA	+	This study
c.1154C>T	Exon 8	Exon 8 skipping	10–20	Sloping to flat	NA	NA	Iran	NA	NA	+	(3)
c.1183G>C	Exon 8	Exon 8 skipping	NA	NA	NA	NA	China	+	+	+	(27)
c.1183G>A	Exon 8	Exon 8 skipping	10–20	Sloping	NA	NA	USA	+	+	+	(3)
c.1183+1G>T	Intron 8	Exon 8 skipping	15–50	Flat	NA	NA	China	+	+	+	(20)
c.1183+1G>C	Intron 8	Exon 8 skipping	20	Sloping to flat	–	–	China	+	+	+	(20)
c.1183+1del	Intron 8	Exon 8 skipping	12–30	Sloping	NA	–	China	NA	NA	+	(16)
			8–30	Sloping	Y	–	China	NA	NA	+	(28)
c.1183+4A>G	Intron 8	Exon 8 skipping	11–50	Sloping	NA	NA	China	–	+	+	(5)
c.1183+5G>A	Intron 8	Exon 8 skipping	NA	NA	NA	NA	Australia	+	+	+	(29)

*In silico* splice prediction tools (SpliceAI, dbscSNV, MaxEntScan) were applied to these variants. Their performance varied, with MaxEntScan achieving 100% accuracy for variants within canonical splice regions, while SpliceAI's accuracy was lower (66.7%) despite its ability to assess all genomic regions (Table 2).

## 4 Discussion

In this study, we report a novel *GSDME* non-sense variant (c.1123G>T; p.Glu375Ter) in a large autosomal dominant deafness Chinese family. To our knowledge, this is the first reported non-sense variant causing DFNA5. This finding directly challenges the long-held notion that non-sense/frameshift variants in *GSDME* are benign due to haploinsufficiency (6–8), compelling a reassessment of how premature termination codons (PTCs) paradoxically lead to gain-of-function (GOF) disorders. The determination of pathogenicity heavily relies on the interpretation of variant, encompassing predictions of the potential consequences resulting from base alterations, which might not always align with biological processes. A multifaceted, comprehensive analysis of variant pathogenicity, leveraging various interpretative dimensions, can aid in averting misdirection stemming from “very strong” evidence.

The present findings serve to further emphasize the consistency of the pathogenic mechanisms of DFNA5. As outlined in our analysis and in previous reports (see Table 2), the vast majority of pathogenic *GSDME* variants, whether involving splice-site alterations in introns 7/8 or missense changes within exon 8, ultimately result in the same pathological outcome: the skipping of exon 8 at the mRNA level. The c.1123G>T variant, despite being predicted as a non-sense variant, has been shown to share the same pathogenic mechanism. Minigene analysis unequivocally demonstrates that it also causes complete skipping of exon 8 (Figure 2). This variant was found to be mechanistically aligned with all other known DFNA5-causing variants, rather than with the presumed loss-of-function (LOF) allele.

Traditionally, non-sense variants and splicing site variants have been viewed as distinct pathogenic pathways. But this non-sense variant unequivocally caused exon skipping. This compelled us to look beyond conventional categories to non-sense-associated altered splicing (NAS), a mechanism that bridges these seemingly separate processes (9, 10). The phenomenon of NAS is characterized by the influence of a PTC on splice site selection, frequently resulting in the exclusion of the exon containing the PTC. This process is believed to be a strategy employed to evade the non-sense-mediated decay (NMD) pathway (11). Bioinformatic analysis suggests that the c.1123G>T nucleotide substitution disrupts a putative binding site for the serine/arginine-rich splicing factor SF2/ASF (an exonic splicing enhancer, ESE) (Figure 2E) (12). The hypothesis is that this weakening of exon definition shifts the balance toward the recognition of a cryptic or competing splice site, resulting in exon 8 skipping. This model elegantly reconciles the non-sense nature of our variant with the established GOF mechanism of DFNA5, providing a specific molecular route by which a PTC can initiate the pathogenic splicing event.

The present study contributes a critical piece of evidence to the GOF hypothesis for DFNA5. It is demonstrated that the pathogenic determinant is not the variant type *per se* (missense or non-sense), but rather its final consequence on pre-mRNA processing. This principle is powerfully illustrated by our querying of population and clinical databases (Genetic Deafness Commons, GDC). As demonstrated in Figure 3B, LOF variants occurring before the critical exon 8 region are typically benign, whereas those that induce exon 8 skipping (e.g., splice-site variants and, as demonstrated in this study, this specific non-sense variant) are pathogenic. This finding provides compelling evidence that refutes the hypothesis of haploinsufficiency and instead highlights the toxic nature of the specific truncated protein resulting from exon 8 loss. This assertion contradicts the notion that the observed effects are merely a consequence of reduced protein dosage.

In accordance with preceding reports on DFNA5, the present pedigree demonstrates substantial inter- and intra-familial phenotypic variability with regard to both age of onset (ranging from the first to third decade) and progression rate (see Table 1) (13). This variability, also observed across different variants (see Table 2), suggests that the core “exon 8 skipping” event, while necessary, may not be sufficient to dictate the precise clinical course (13). The expression of disease is likely to be modulated by modifying genetic factors, environmental influences (e.g., noise), or stochastic processes in the cochlea. From a clinical diagnostic perspective, this variability underscores the necessity of prioritizing functional splicing assays over phenotype-based predictions when assessing *GSDME* variants in the exon 8 critical region.

It is important to acknowledge the limitations of the present study. The minigene assay, while widely regarded as the gold standard for splicing analysis, is an *in vitro* model (PMID: 35716007). The NAS mechanism would be further validated by additional research using patient-derived cells or knock-in animal models *in vivo*, which would also make it easier to examine the downstream cytotoxic effects unique to this non-sense variant. Furthermore, the genetic modifiers underlying the observed phenotypic spectrum remain to be discovered. Finally, we have discovered and described the first pathogenic non-sense variant in *GSDME*'s exon 8. The present study not only expands the variantal and mechanistic spectrum of DFNA5 by implicating NAS, but also fundamentally refines the genetic counseling framework for *GSDME*. It is now imperative that variants in this region are assessed for their splicing impact, regardless of their predicted protein-level effect. This finding serves to reinforce the unifying principle that DFNA5 pathogenesis converges exclusively on the gain-of-function mechanism triggered by exon 8 skipping.

## 5 Conclusion

In this study, we successfully identified a novel non-sense variant in the exon 8 of *GSDME* in a Chinese family with hereditary deafness. This variant causes NAS in exon 8, which was found to be causative for deafness. To date, this is the first report of a *GSDME* non-sense mutation leading to DFNA5, expanding the spectrum of *GSDME* mutations and phenotypes, and further confirming the pathogenic mechanism of *GSDME* as gain-of-function.

## Data Availability

The original contributions presented in the study are included in the article/Supplementary material, further inquiries can be directed to the corresponding author.
