# Supplementary figures and images for: A novel non-sense variant in GSDME causing exon skipping associated with DFNA5 in a large Chinese family
Source: Front Neurol. 2026 Feb 6;17:1752843. doi: 10.3389/fneur.2026.1752843 (PMC12920056; doi:10.3389/fneur.2026.1752843)

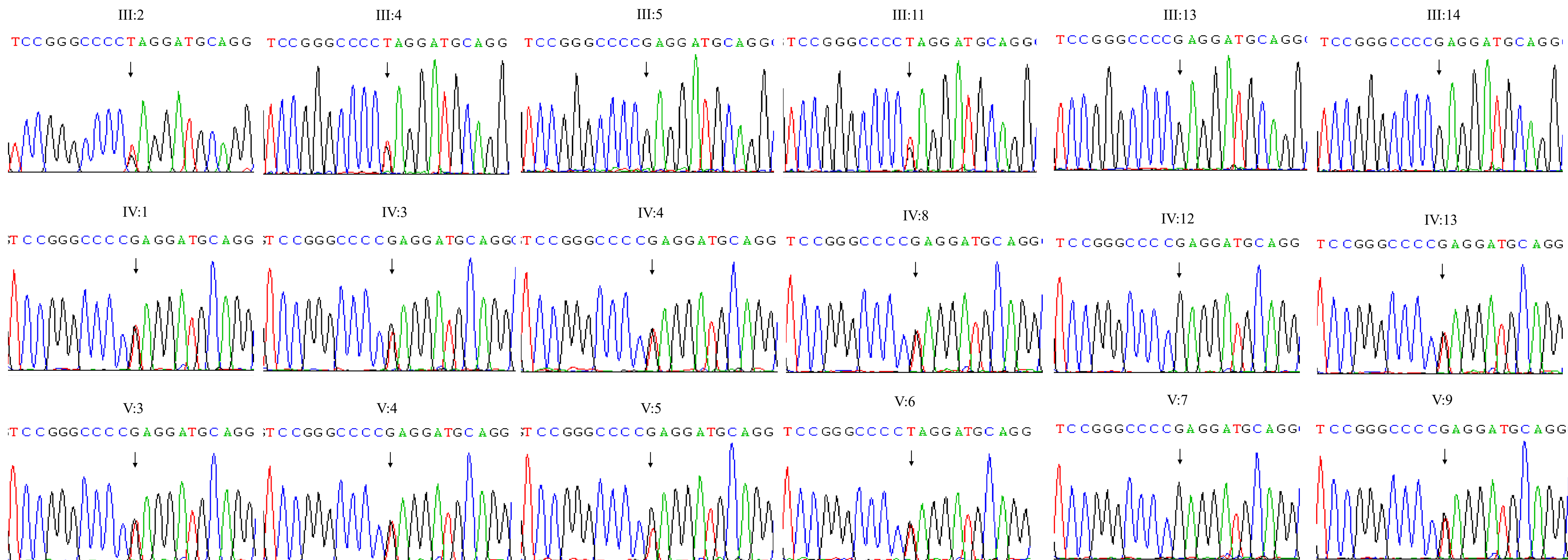

Supplement: Supplementary file 1 [file Data_Sheet_1.pdf]
